# The Association Between Commonly Investigated User Factors and Various Types of eHealth Use for Self-Care of Type 2 Diabetes: Case of First-Generation Immigrants From Pakistan in the Oslo Area, Norway

**DOI:** 10.2196/publichealth.7009

**Published:** 2017-10-05

**Authors:** Naoe Tatara, Hugo Lewi Hammer, Hege Kristin Andreassen, Jelena Mirkovic, Marte Karoline Råberg Kjøllesdal

**Affiliations:** ^1^ Department of Computer Science Faculty of Technology, Art and Design Oslo and Akershus University College of Applied Sciences Oslo Norway; ^2^ Centre for Care Research Norwegian University of Science and Technology Gjøvik Norway; ^3^ Norwegian Centre for E-health Research University Hospital of North Norway Tromsø Norway; ^4^ Center for Shared Decision Making and Collaborative Care Research Oslo University Hospital Oslo Norway; ^5^ Department of Community Medicine and Global Health Institute of Health and Society, Faculty of Medicine University of Oslo Oslo Norway

**Keywords:** immigrants, type 2 diabetes, self-care, information seeking behavior

## Abstract

**Background:**

Sociodemographic and health-related factors are often investigated for their association with the active use of electronic health (eHealth). The importance of such factors has been found to vary, depending on the purpose or means of eHealth and the target user groups. Pakistanis are one of the biggest immigrant groups in the Oslo area, Norway. Due to an especially high risk of developing type 2 diabetes (T2D) among this population, knowledge about their use of eHealth for T2D self-management and prevention (self-care) will be valuable for both understanding this vulnerable group and for developing effective eHealth services.

**Objective:**

The aim of this study was to examine how commonly were the nine types of eHealth for T2D self-care being used among our target group, the first-generation Pakistani immigrants living in the Oslo area. The nine types of eHealth use are divided into three broad categories based on their purpose: information seeking, communication, and active self-care. We also aimed to investigate how sociodemographic factors, as well as self-assessment of health status and digital skills are associated with the use of eHealth in this group.

**Methods:**

A survey was carried out in the form of individual structured interviews from September 2015 to January 2016 (N=176). For this study, dichotomous data about whether or not an informant had used each of the nine types of eHealth in the last 12 months and the total number of positive answers were used as dependent variables in a regression analysis. The independent variables were age, gender, total years of education, digital skills (represented by frequency of asking for help when using information and communication technology [ICT]), and self-assessment of health status. Principal component analyses were applied to make categories of independent variables to avoid multicollinearity.

**Results:**

Principal component analysis yielded three components: *knowledge*, comprising total years of education and digital skills; *health*, comprising age and self-assessment of health status; and *gender*, as being a female. With the exception of closed conversation with a few specific acquaintances about self-care of T2D (negatively associated, *P*=.02) and the use of ICT for relevant information-seeking by using search engines (not associated, *P*=.18), the *knowledge* component was positively associated with all the other dependent variables. The *health* component was negatively associated with the use of ICT for closed conversation with a few specific acquaintances about self-care of T2D (*P*=.01) but not associated with the other dependent variables. *Gender* component showed no association with any of the dependent variables.

**Conclusions:**

In our sample, knowledge, as a composite measure of education and digital skills, was found to be the main factor associated with eHealth use regarding T2D self-care. Enhancing digital skills would encourage and support more active use of eHealth for T2D self-care.

## Introduction

### Factors Associated With Electronic Health (eHealth) Use

In the last decade, we have seen a rapid development of accessible information and communication technology (ICT). The cost of accessing the Internet has decreased, especially mobile broadband, and there has been an increase in the variety of services and products for personal self-care. Commensurate with this trend, the use of electronic health (eHealth) has become a general practice in many developed countries. Purposes for using eHealth for self-care include, for example, seeking related information [1-10], Web-based communication with health care experts or peers [9,11-13], and keeping track of user’s health information or self-assessment for reflection on self-care [5,9,14,15]. These purposes are common for both prevention and self-management of various types of diseases, especially lifestyle-related chronic diseases. The World Health Organization [16] defines self-care as keeping health, prevention of, and dealing with illness. In this paper, we use the term “self-care” as an all-embracing definition that includes both self-management and prevention of illness.

A large number of studies have explored how different factors are associated with target users’ eHealth use for self-care, both in general and with focus on chronic diseases [5,7,9,10,14,15,17-46]. Factors such as demographic information (including age, gender, and education level) are often investigated as independent variables of eHealth use. Health-related factors are also often used as independent variables, but such factors vary considerably depending on the purpose of the study. Knowledge about the association between user factors and eHealth use is vital when designing and developing new eHealth services for similar target users.

We reviewed relevant literature that were published within the last 5 years and that analyzed data obtained in 2010 or later. We limited ourselves to this time frame for two reasons: one is the rapid evolution of mobile phone technology in the years preceding 2010, and the other is the time it would have taken until published literature reflected such changes. Moreover, some studies have found that the factors influencing the use of eHealth changed over time. By comparing data from 2005, 2007, and 2012, Bjunowska-Fedak [21] showed that, in Poland, the association between gender and Internet use for health-related information has switched over the years. McCully et al [47] also found that in the United States, those using the Internet to help with diet, weight, and physical activity had become “younger, less educated, and more likely to be female and single in 2011 than in 2007.”

Tables 1-3 show summaries of studies exploring the use of eHealth for different purposes. Although a number of studies do not distinguish types of Internet use and include a very broad purpose related to health care and illness, we sorted studies and findings depending on their purpose and means where possible. The tables show how education level, age, and being a female are associated with the use of eHealth in each study. Multimedia Appendix 1 shows a complete summary of each study shown in Tables 1-3.

**Table 1:** Studies investigating Web-based health information–seeking and associating factors.

Author (year)	Description of eHealth^a^ use	Education level	Age (years)	Female
Kalantzi et al, 2015 [29]	Using the Internet as an important source for information about diabetes	(+)^b^	(−)^c^	NS^d^
Lee et al, 2012 [31]	Using the Internet to seek health or medical information	(+)	(−)	None^e^
Mesch et al, 2012 [36]	Frequency of searching for health information on the Internet	(−) College, graduate school	(−)	(+)
Gonzalez et al, 2016 [24]	Health information–seeking behavior in the last 12 months	(+)	(−) >65	(+)
Wangberg et al, 2015 [9]	Experience in reading about diet and exercise on the Web	(+)	None	(+)
Manierre et al, 2015 [34]	(Among Internet users) Experience in looking for health information on the Internet for self or someone else in the past 12 months	None	None	(+)
Lee et al, 2014 [48]	Experience in either of the following in the last 12 months: Participating in an online support group for people with similar health or medical issues Using email or the Internet to communicate with a doctor or doctor's office Using the Internet to look up health or medical information	(+)	NS	NS
Kontos et al, 2014 [5]	Using the Internet to download health-related information to a mobile device in the last 12 months	(+) College degree or more versus some college	NS	NS
	Using the Internet to look for health or medical information for self in the last 12 months	NS	(−)	NS
AlGhamdi et al, 2015 [17]	Using the Internet to search for health-related information	(+)	Inconsistent (see Multimedia Appendix 1 for details)	(+)
Bjunowska-Fedak et al, 2015 [20]	Using the Internet to obtain information about health or illness	(+)	(−) Sample age above 60	NS
Bjunowska-Fedak, 2015 [21]	Using the Internet to get information about health or illness at least once a year (including using interactive Internet health services)	NS	(−)	(+)
Duplaga et al, 2013 [23]	Declaration of the Internet as one of main sources of health-related information	(+)	(−)	NS
Beck et al, 2014 [18]	Having used the Internet to look for information or advice about health during the past 12 months	None	(+) Sample age 15-30	Conditionally (+) those with psychological distress, being pregnant, or having a child or more
Nölke et al, 2015 [38]	Using the Internet to search for information on medical or health issues	(+) On the basis of social class index comprising of “educational qualification,” “occupational status,” and “household net income”	NS	(+)

^a^eHealth: electronic health.

^b^(+) indicates positive association with eHealth use (*P*<.05).

^c^(−) indicates negative association with eHealth use (*P*<.05).

^d^NS: not significant; no significant association with eHealth use.

^e^None: the factor was not investigated in a study.

**Table 2:** Studies investigating Web-based communication with experts or peers about health and associating factors.

Author (year)	Description of eHealth^a^ use	Education level	Age (years)	Female
Mesch et al, 2012 [36]	Frequency of participating in Internet forums about health issues or sent an email to a physician or a nurse	NS^b^	NS	NS
Wangberg et al, 2015 [9]	Experience in asking questions about exercise or diet to experts	(−)^c^	None^d^	NS
	Experience in posting a status about exercise or diet on a social networking site	(+)^e^	None	(−)
	Experience in sharing exercise or diet data with others online	NS	None	(+)
	Experience in discussing exercise or diet with peers	NS	None	NS
Kontos et al, 2014 [5]	Experience of using email or the Internet to communicate with a doctor or doctor's office in the last 12 months	(+) College degree or more versus high school degree or less	(−) Age between 18-34 versus >65	(+)
	Experience of participating in an online support group for people with a similar health or medical issue in the last 12 months	NS	NS	(+)
	Experience of visiting a social networking site to read and share about medical topics in the last 12 months	Inconsistent	(−) Age between 18-34 versus >65; and age between 35-49 versus >65	NS
Tennant et al, 2015 [42]	Having used the Internet for any of the following reasons to locate or share health information in last 12 months: (1) participated in a Web-based support group, (2) used a social networking site such as Facebook, Twitter, or LinkedIn, or (3) wrote in a Web-based diary or blog	(+) 4 years of college or more versus less than high school	NS (sample: age >50)	(+)
Thackeray et al, 2013 [43]	Using a social networking site (SNS) for health-related activities	NS	(−)	(+)

^a^eHealth: electronic health.

^b^NS: not significant; no significant association with eHealth use.

^c^(−) indicates negative association with eHealth use (*P*<.05).

^d^the factor was not investigated in a study.

^e^(+) indicates positive association with eHealth use (*P*<.05).

Most of the studies concentrate on experiences of Web-based health information search. The number of studies investigating Web-based communication or use of Web applications is still limited. In general, having a high education, being young, and being female are positively associated with eHealth use in many of the studies listed. However, a few studies ([36] in Table 1, [9] regarding “Experience in asking questions about exercise or diet to experts” in Table 2, and [9] regarding “Experience in posting a status about exercise or diet on a social networking site” in Table 2) found associations opposite to this. Moreover, some studies found inconsistent or no significant association between these factors and eHealth use, especially regarding Web-based communication with experts or peers (see Tables 1-3).

In addition to the three factors above, health-related factors and socioeconomic status are also commonly examined for their association with eHealth use. Examples of health-related factors include subjective measure of being in good health [9,15,20,21,29-31,38,42,43] and diagnosis of a chronic disease, either a nonspecific disease [14,17,18,20,21,24,38] or a specific disease type [5,23,29,30,36]. Socioeconomic status is most typically expressed by income and income-based variables [14,17,22,24,29,32,34,36,42,43,49]. Associations between these factors and use of eHealth do not seem as consistent as education, age, and gender (see Multimedia Appendix 1).

**Table 3:** Studies investigating the use of mobile apps or Web applications for active self-care and associating factors.

Author (year)	Description of eHealth^a^ use	Education level	Age (years)	Female
Krebs et al, 2015 [14]	Experience of having ever downloaded an “app” to track anything related to a user's own health	(+)^b^ (Ref less than high school)	(−)^c^	NS^d^
Bender et al, 2014 [15]	Experience of downloading health apps	(+) Ref high school	(−)	NS
Wangberg et al, 2015 [9]	Using Internet- or mobile-based programs to support health behavior	NS	None^e^	(+)
	Keeping a Web-based exercise or diet journal	(+)	None	(+)
Kontos et al, 2014 [5]	Experience of using the Internet to keep track of personal health information in the last 12 months	(+) College degree or more versus high school degree or less	NS	(+)
	Experience of using a website to help with diet, weight, or physical activity in the last 12 months	(+)	(−)	NS

^a^eHealth: electronic health.

^b^(+) indicates positive association with eHealth use (*P*<.05).

^c^(−) indicates negative association with eHealth use (*P*<.05).

^d^NS: not significant; no significant association with eHealth use.

^e^None: the factor was not investigated in a study.

Interestingly, few studies have investigated the relationship between factors relevant to digital skills and eHealth use. Lee et al [30] found that the use of social network services (SNS) is positively associated with eHealth use among Hispanic adults in Northern Manhattan. Here, eHealth use was seen as having an experience of either (1) participating in an online support group for people with similar health or medical issues, (2) using email or the Internet to communicate with a doctor or doctor’s office, or (3) using the Internet to look up health or medical information. It is therefore unclear which of these three types had a significant association with SNS use. Bender et al [15] carried out a survey by letting informants choose either a Web-based form or paper form. They found that taking the paper survey was negatively associated with experience of downloading health apps compared with having taken a Web-based survey.

### A Case: First-Generation Immigrants From Pakistan in Norway and eHealth Use

Norway is one of the most highly digitalized countries in Europe. As of 2016, 96% of the population has access to the Internet, and 75% have basic digital skills [50]. The government promotes digitalization of public services, including a health and care service portal with self-service solutions [51]. The national health and care service portal is growing in use [51,52]. In 2015, 62% of the population in the age range of 16 and 79 years had searched the Internet for health-related information in the last 3 months [4]. However, it is not yet clear whether the same applies to specific populations of different demographic makeups. For example, several recent studies show that being an immigrant is a negatively associated factor of eHealth use for certain purposes within self-care [24,36,38,45]. On the other hand, ethnicity is not consistently associated with eHealth use [14,41,47,53-56]. Two studies in the United States observed changes in association between ethnical background and eHealth use over time. McCully et al [47] found that compared with non-Hispanic whites, the proportion of users of the Internet for diet, weight, and physical activity decreased among non-Hispanic blacks, whereas it increased among Hispanics from 2007 to 2011. Wilson et al [57] found that more Hispanics used online support groups in 2012 than in 2007.

Pakistani immigrants are one of the large immigrant groups in the Oslo area [58]. Previous studies have shown that they have an alarmingly high prevalence rates of diabetes (women: 26.4%, men: 20.0%) compared with ethnic Norwegian population (women: 2.7%, men: 6.4%) [59], in addition to having relatively low ICT skills [60]. These two factors make this group of particular interest with regard to the use of eHealth for self-care of type 2 diabetes (T2D). We should note that the referred study [59] reports the prevalence rate of diabetes without dividing diabetes into type 1 diabetes (T1D) and T2D. However, the prevalence rate of T1D of the Pakistani population is considered to be much lower than that of ethnic Norwegian population [61]. Regarding the ICT skills, the only source of information on ICT skills of the first-generation immigrants from Pakistan in Norway is a survey [60] published in 2010. Since then, Smartphones have become much more popular, and more and more people have access to ICT. Moreover, there is a distinct lack of contemporary literature on eHealth use for self-care among the immigrant population in European countries that is from outside Europe [45]. Investigating the use of eHealth in relation to ICT skills and other relevant factors will give a new and updated insight of the potential for and barriers to benefit from eHealth in this population.

### Objectives

From a larger survey among first-generation Pakistani immigrants in Oslo, this paper focuses on results regarding the use of eHealth for self-care of T2D depending on its purpose and means, which are categorized as follows: (A) For seeking T2D-relevant information: (a) by using search engines that require input of search terms, (b) on specific websites or by email subscriptions that can be navigated by only scrolling and clicking, or (c) by searching for software programs on personal computers, or apps on a mobile phone or a tablet (mobile apps) that could be used as a look-up tool; (B) For communicating or consulting about T2D self-care: (d) by using ICT in general for closed conversation with a few specific acquaintances such as voice or video or text communication, (e) via SNS, (f) on portals for peer communication, or (g) by online consulting with experts in diabetes; (C) For active decision making on T2D self-care (h) by using Web applications for or mobile apps tracking health information such as diet, physical activities, weight, blood glucose level, and so on, or (i) by using Web applications or mobile apps to assess one’s own health status with regard to T2D.

The objective of this paper is to answer the following research questions: (1) How common is eHealth use for T2D self-care among first-generation immigrants from Pakistan in the Oslo area? and (2) To what extent are education, age, gender, digital skill, and self-assessed health associated with the use of eHealth for self-care of T2D in this population?

## Methods

### Description of Survey

We carried out the survey from September 2015 to January 2016. Ethical approval was given to the project protocol by Norwegian Social Science Data Services in June 2015 (project number: 43549). We employed purposive sampling for the recruitment of informants. Reflecting the results from our pilot, the following inclusion criteria were set, as shown in the survey protocol paper [62]: (1) immigrated from Pakistan after the age of 18 years; (2) live in the Oslo area; (3) speak Urdu (the official language of Pakistan) as the primary language in their private life; (4) aged between 25 and 59 years; (5) have access to or interest in ICT tools (personal computer, tablet, or smartphone), connected to the Internet in daily life; and (6) motivated for and capable of performing activities for self-care of T2D.

On the basis of the recommendations on recruitment in immigrant populations [63] and experience from previous studies that included Pakistani immigrants in Norway [64-67], we used a multirecruitment strategy. This included recruiting informants first via an already established network of 2 research assistants in the target group, approaching new potential informants in the local community, and snowball sampling [68]. In total, 176 informants participated in the survey.

The survey employed individual structured interviews. The research assistants, who are fluent in speaking Urdu, interviewed the informants and recorded their verbal responses. The protocol of the whole survey, as well as the entire set of questions used in the survey, can be found elsewhere [62].

### Variables

We chose to analyze the following variables to be able to compare eHealth use in our survey sample with relevant studies shown in Tables 1-3.

#### Independent Variables

Demographic variables include being a female, age group by a range of birth year, and the total years of education from Pakistan and Norway. The decision to use age group by a range of birth year rather than exact age was made to avoid a potential risk that, because of the relatively small sample size, an informant could be identified by a combination of the answers provided to some questions in the survey [68]. To enable dealing with age group as one variable, we used the middle year of each range as the representative year of birth of each group.

In Pakistan, primary education lasts for 5 years, starting from the age of 5. This is followed by a 3-year junior secondary education after which there is 2-year secondary school and then 2-year higher secondary school before undergraduate level [69]. In case an informant had education in Norway, we added the number of completed years of education to the years of education taken in Pakistan.

Self-assessment of health status was obtained by a multiple-choice question with answer alternatives being “Excellent (5),” “Very good (4),” “Good (3),” “Fair (2),” “Going up and down (1),” and “Poor (0)” based on a question used in [70]. The question was associated with health status in general. Nevertheless, we considered this variable to be potentially highly relevant with self-care of T2D, given their interest in this disease as one of the inclusion criteria.

Lack of digital skills were captured by a question related to the frequency of asking for assistance when using ICT devices, with answer alternatives being “Always (4),” “Often (3),” “Sometimes (2),” “Seldom (1),” and “Never (0).” In the analysis, these two variables were treated as continuous data as they present ordinal categorical data with more than five categories [71].

#### Dependent Variables

As dependent variables, we used dichotomous answers to the nine questions asking about whether or not informants had used eHealth for self-care of T2D in the last 12 months, depending on the purpose and means described in the Objective subsection. We also used a total number of positive answers to these nine questions as a variable showing how a wide variety of eHealth activities an informant was engaged with for self-care of T2D.

### Statistical Analysis

Logistic regressions were used to assess associations between experience with each purpose of eHealth use (dependent variable) and the independent variables. The variety of eHealth use can be interpreted as a count variable, therefore, we resorted to Poisson regression.

Although we were interested in digital skills as a separate factor from education level, it is probable that they are correlated to each other. Given the result of a report about digital skills of immigrants with a Pakistani background in Norway, it is also probable that the age and gender would be also highly correlated to digital skills [60]. To avoid multicollinearity, we used the principal components of the independent variables. Three principal components were selected, based on the scree plot [72], and we used Varimax rotation [73]. Variables with a high score in absolute value were used to characterize each principal component. Principal component scores were computed, and the scores were used as the independent variables in regression analyses.

## Results

### Characteristics of the Sample and eHealth Use

Table 4 shows the distribution of the informants by each variable. Male informants (n=42) were considerably fewer than female informants (n=134). However, the informants were similarly distributed by age group for both genders. In total, 80 informants (45.5%, 80/176) had up to 10 years of education; 28 informants (15.9%, 28/176) had only up to 5 years of primary education—they were all women. On the other hand, 42 female informants, which is nearly one-third of the female sample, had completed college education or higher. In total, 12 informants had been to a Norwegian school, among which 7 informants had been to college level or higher.

The majority of the informants answered that their health status, in general, was good or better. One who answered “going up and down” mentioned explicitly that the condition was due to pregnancy.

Forty-four informants (25.0%, 44/176) reported that they need assistance in the use of ICT tools always or often. On the other hand, 68 informants (38.6%, 68/176) expressed they did not need any assistance in the use of ICT tools.

Regarding eHealth use for information seeking, 63 informants (35.8%, 63/176) reported use of portals and similar sources that required only simple operations such as scrolling and clicking (b). On the other hand, no more than 35 informants (19.9%, 35/176) reported the use of a search engine that requires text input (a). Only 8 informants (4.5%, 8/176) had searched the Internet for mobile apps or software programs on personal computers for look-up of relevant information to T2D self-care (c).

Eighty-four informants (47.7%, 84/176) had used ICT for closed communication with acquaintances regarding self-care of T2D (d), and 58 informants (33.0%, 58/176) had used SNS for communication with others about self-care of T2D (e). However, only 9 informants reported using portals for peer communication in the context of T2D self-care (f). There was only 1 informant who had asked about issues relevant to T2D self-care to an expert on the Web (g). Mobile apps and Web applications for active decision making on T2D self-care were not very popular. Twenty-five informants (14.2%, 25/176) reported the use of mobile apps or Web applications for keeping track of health information (h), 38 informants (21.6%, 38/176) used mobile apps or Web applications for self-assessment of health status (i), and 41 informants (23.2%, 41/176) had never used ICT for T2D self-care. Among the 46 informants (26.1%, 46/176) who had used only one type of eHealth in the context of T2D self-care, 31 informants (17.6%, 31/176) answered that they were using ICT for closed communication with acquaintances (d). No informant had experience of eight or more types of eHealth for T2D self-care.

### Association Between User Factors and eHealth Use

Table 5 shows the computed principal components. We see that the total years of education (positive value) and the frequency of asking for help (negative value) dominate the first principal component (PC1). Education means acquiring knowledge, whereas asking for help indicates lack of knowledge. We therefore refer to this principal component as *knowledge* below.

The second principal component (PC2) is strongly related to health status (positive value) and to some extent to age (negative value). The self-assessment of health status and age were negatively correlated according to Pearson correlation test that was separately applied to these variables (correlation=−.32, *P*<.001). This result is reasonable, given that health status in general declines with age. Therefore, we refer to this principal component as *health*.

The third principal component (PC3) is related to *gender*.

**Table 4:** Descriptive characteristics of the survey informants (N=176).

Variables			Informants, n (%)
**Gender**			
	Male		42 (23.9)
	Female		134 (76.1)
**Age group by birth year range**			
	1981–1990		54 (30.7)
	1971-1980		61 (34.7)
	1956-1970		61 (34.7)
**Total years of education from Pakistan and Norway**			
	0 years		14 (8.0)
	5 years		13 (7.4)
	<10 years		17 (9.7)
	<12 years		33 (18.8)
	<14 years		39 (22.2)
	14 years or more		55 (31.3)
**Self-assessment of health status (score)**			
	Excellent (5)		11 (6.3)
	Very good (4)		27 (15.3)
	Good (3)		70 (39.8)
	Fair (2)		37 (21.0)
	Going up and down (1)		19 (10.8)
	Poor (0)		12 (6.8)
**Frequency of asking for help when using ICT^a^**			
	Always (4)		18 (10.2)
	Often (3)		26 (14.8)
	Sometimes (2)		51 (29.0)
	Seldom (1)		12 (6.8)
	Never (0)		68 (38.6)
**Experience of eHealth^b^** **use for T2D^c^** **self-care in the last 12 months**			
	**(A) For seeking relevant information**		
		(a) By using search engines that require input of search terms	35 (19.9)
		(b) On specific websites or by mail subscriptions that can be navigated by only scrolling and clicking	63 (35.8)
		(c) By searching for software programs on personal computers or applications on mobile phone or tablet (mobile apps) that could be used as a look-up tool	8 (4.5)
	**(B) For communication and consulting**		
		(d) By using ICT in general for closed conversation with a few specific acquaintances	84 (47.7)
		(e) By social networking sites	58 (33.0)
		(f) On portals for peer communication	9 (5.1)
		(g) By online consulting with experts in diabetes	1 (0.6)
	**(C) For active decision making on self-care by using Web applications or mobile apps for**		
		(h) Keeping track of health information	25 (14.2)
		(i) Self-assessment of health status	38 (21.6)

**Total number (variety) of eHealth types experienced**			
	8 or more		0 (0.0)
	7		2 (1.1)
	6		5 (2.8)
	5		7 (4.0)
	4		9 (5.1)
	3		28 (15.9)
	2		38 (21.6)
	1		46 (26.1)
	0		41 (23.3)

^a^ICT: information and communication technology.

^b^eHealth: electronic health.

^c^T2D: type 2 diabetes.

**Table 5:** Computed principal components (PCs).

Variables	PC1^a^	PC2	PC3
Being a female	−.24	−.15	.87
Age	−.41	−.58	−.49
Total years of education	.85	.13	−.08
Self-assessment of health status	.17	.90	−.21
Frequency for asking help when using ICT^b^	−.82	−.23	.15

^a^PC: principal component.

^b^ICT: information and communication technology.

Table 6 refers to the results of the regression analyses. The analysis included all the dependent variables presented in the Methods section excluding (g), that is, online consulting with experts in diabetes because only one informant had such an experience.

The *knowledge* component is strongly and positively related to the total number (variety) of eHealth experience types and all the dichotomous dependent variables, with the exception of two variables: (1) closed online communication about T2D with a few acquaintances (d), which is negatively related and (2) seeking relevant information by using search engines that require input of search terms (a), which is unrelated. The *health* component is negatively related to closed online communication about T2D with a few acquaintances (d), and there is an indication of a positive relation between the *health* component and the use of Web applications and mobile apps for active decision making on T2D self-care by tracking of health information (*P*=.05). The *gender* component has no significant association with any of the dependent variables.

**Table 6:** Result of regression analyses.

Variables			Estimate	Standard error	z value	*P* value
**(A) For seeking relevant information**						
	**(a) By using search engines that require input of search terms**					
		Intercept	−1.415	0.194	−7.299	<.001
		Knowledge	0.282	0.207	1.358	.18
		Health	0.002	0.192	0.011	>.99
		Gender	−0.137	0.181	−0.754	.45
	**(b) On specific websites or by email subscriptions that can be navigated by only scrolling and clicking**					
		Intercept	−0.615	0.164	−3.750	<.001
		Knowledge	0.489	0.179	2.734	.006
		Health	0.093	0.163	0.571	.57
		Gender	−0.082	0.157	−0.522	.60
	**(c) By searching for software programs on personal computers or applications on mobile phone or tablet (mobile apps) that could be used as a look-up tool**					
		Intercept	−3.955	0.706	−5.602	<.001
		Knowledge	1.298	0.650	1.996	.046
		Health	0.518	0.440	1.177	.24
		Gender	0.745	0.517	1.441	.15
**(B) For communication and consulting**						
	**(d) By using ICT^a^in general for closed conversation with a few specific acquaintances**					
		Intercept	−0.084	0.158	−0.531	.60
		Knowledge	−0.375	0.160	−2.341	.02
		Health	−0.400	0.163	−2.454	.01
		Gender	0.231	0.159	1.450	.15
	**(e) By social networking sites**					
		Intercept	−0.766	0.171	−4.487	<.001
		Knowledge	0.597	0.191	3.120	.002
		Health	0.068	0.168	0.406	.69
		Gender	−0.015	0.161	−0.092	.93
	**(f) On portals for peer communication**					
		Intercept	−3.329	0.481	−6.928	<.001
		Knowledge	0.988	0.500	1.976	.048
		Health	−0.132	0.361	−0.364	.72
		Gender	0.298	0.378	0.788	.43
**(C) For active decision making on T2D^b^self-care by using Web applications or mobile apps for**						
	**(i) Self-assessment of health**					
		Intercept	−1.640	0.249	−6.597	<.001
		Knowledge	1.165	0.289	4.036	<.001
		Health	0.312	0.206	1.515	.13
		Gender	0.096	0.189	0.509	.61
	**(h) Keeping track of health information**					
		Intercept	−2.309	0.334	−6.922	<.001
		Knowledge	1.257	0.365	3.447	<.001
		Health	0.487	0.251	1.942	.05
		Gender	0.039	0.218	0.181	.86
**Total number (variety) of eHealth types experienced**						
		Intercept	0.566	0.582	9.725	<.001
		Knowledge	0.290	0.063	4.644	<.001
		Health	0.024	0.057	0.420	.68
		Gender	0.046	0.055	0.823	.41

^a^ICT: information and communication technology.

^b^T2D: type 2 diabetes.

## Discussion

### Principal Findings and Implications For Future Studies

This study targeted first-generation immigrants from Pakistan living in the Oslo area and examined their use of various types of eHealth in the context of T2D self-care. As there has not been any data showing eHealth use by this target population, this study increased our understanding of one of the biggest minority groups in Norway. Wilson et al [57] argue that “it can be misleading to study use of aggregated eHealth services within portals, as results are likely to mask true usage patterns of the distinct services.” By asking about the use of eHealth depending on its purpose and means, this study could highlight how and for what the target group use eHealth for T2D self-care, as well as the difference in how user factors are associated with use of eHealth.

The finding that nearly half of the survey sample has used ICT for closed communication with acquaintances about T2D self-care implies that T2D self-care is not a rarity among the target population. This is reasonable, considering the high prevalence of diabetes among the target population [59]. This is also supported by the findings that more than one-third of the sample has used SNS for communicating or consulting about T2D self-care. A low proportion of the informants had an experience in peer communication or online consulting with experts regarding T2D self-care, which is in line with findings in similar studies [5,9,31]. Despite the common awareness and high attention to T2D self-care, our findings indicated that individuals in this population sample have not yet taken much advantage of ICT for active self-care for keeping track of health or self-assessment of health status.

To the authors’ best knowledge, there is no other similar study investigating eHealth use concerning T2D self-care among the ethnic Norwegian population. The closest is the study by Wangberg et al [9], based on a Web-based survey to a sample of 1028 informants (aged above 15 years) registered as Web panel, shown in Tables 1-3. The study was about diverse types of eHealth use, with a focus on diet and physical activity. Due to the difference in questions and inclusion criteria of the informants, the studies cannot be directly compared. However, the proportion of users of each type of eHealth in our sample seems less than that of their sample (Multimedia Appendix 1). More research investigating samples from ethnic Norwegian populations who are interested in and capable of T2D self-care is required to investigate whether there are any gaps between the two target user groups.

As shown in Table 1, many relevant studies do not specify the means for Web-based health information seeking. Nevertheless, based on the literature review, which showed that most studies have found a positive association between education level and Web-based health information seeking and the nature of search engines, we expected that the principal component *knowledge* would have a positive association with use of search engines for relevant information about T2D self-care. However, the *knowledge* component was not associated with use of search engines in our study. Rather, it was positively associated with use of ICT-based T2D self-care information resources that can be used by simple operations. In our study, a larger proportion of participants had used Web portals and similar resources that require only simple operations compared with search engines, for seeking relevant information about T2D self-care. Search engines are normally used for exploring a specific topic or question. The fact that T2D is a common topic among the target population may partly explain the low percentage of the informants having attempted to use search engines to seek and explore in depth relevant information to T2D self-care. We need a further analysis of the whole survey data, including language proficiency and preference as to ICT use and for the purpose of T2D self-care, to understand the finding.

The principal component *knowledge* was differently associated with use of ICT for communicating or consulting about T2D self-care depending on its means and who they communicate with (Table 6). Studies listed in Table 2 also show that education level is not a consistently associated factor of such use. The *knowledge* component was strongly associated with the search of software and the use of Web applications or mobile apps. As *knowledge* component is strongly related to education level, this result is in line with the studies listed in Table 3. At the same time, the *knowledge* component includes the independent variable related to digital skills. Implication here is positive because enhanced digital skills may have a potential to engage the target population with use of eHealth for active T2D self-care. Cultivating digital skills by providing courses, for example, would be a more reasonable solution than increasing the total years of formal education.

In this study, the principal component *health* was found to have a negative association with use of ICT for closed communication with a few acquaintances about T2D self-care. Other than this, the *health* component did not have any associations with purpose or mean of eHealth use. The principal component *health* had strong positive loadings for self-assessment of health status and negative loadings for age. The implication here could be that the older and less healthy people are more likely to use Web-based conversations with their acquaintances regarding T2D self-care compared with younger and healthy people, but otherwise, users of eHealth for T2D self-care in this sample are varied in age and health status. However, all the three principal components had quite similar level of loadings for age. Given that age was very often negatively associated with eHealth use in relevant studies, whereas self-assessment of health status was not as often correlated to eHealth use as age, it might be worth looking at age as an independent factor in a future study with a larger sample.

### Limitations

Due to the existing privacy and security regulation, use of the middle year of each range of age group as the representative year of birth was the best possible solution within the choices we could make. However, the results might have changed slightly if we instead could have used the actual age of each informant in the analyses.

The study sample had uneven gender balance, which may not reflect the gender balance of the population that fulfills our inclusion criteria. The 2 research assistants reported several cases where they failed to recruit male informants because of them falling outside one of the inclusion criteria or their unwillingness to participate. The cases included immigration to Norway at the age of 16 or 17 years, which caused exclusion because of one of the inclusion criteria. We set an inclusion criterion that informants had immigrated to Norway after age of 18 years because of the age authorized as a legal adult in Norway. In Pakistan, age at completion of higher secondary education can be 17 years. If our inclusion criterion regarding the age at immigration had been 16 years, we might have been able to include more male informants. The other cases are negative attitudes toward being asked about personal health, engagement in night-shift work and sleeping during the daytime, and a negative reaction to interaction with the female research assistants. For future similar studies, using male research assistants may also help to ensure recruiting male informants. Due to the limited budget of the project and cost ineffectiveness of purposeful sampling, we needed to focus on recruiting as many informants as possible, instead of focusing on including more males. Gender was controlled for in the regression analysis to account for the imbalance.

In a survey about digital skills of immigrant groups in Norway [60], 40 concrete questions were used to measure informant’s digital skills. We considered that the number is too large and many topics covered in the 40 questions were irrelevant to the survey. Therefore, we decided to use frequency of asking for help as a subjective measure of digital skills. This is one possible limitation because the question may not be able to reflect objectively measured digital skills.

### Conclusions

This study adds to the knowledge about the use of eHealth for T2D self-care among first-generation Pakistani immigrants living in the Oslo area, especially those who are interested in and capable of T2D self-care. *Knowledge* as a composite measure of education and digital skills was related to the use of eHealth for self-care of T2D, except for seeking relevant information by using search engines. *Health*, as a composite measure of self-assessment of general health status and younger age, was negatively related to use of closed online communication services with acquaintances about T2D self-care. Otherwise, neither *health* nor *gender* was considered to be an important factor associated with eHealth use for T2D self-care. Pakistani immigrants in the Oslo area are using eHealth for active self-care of T2D to a limited extent. Providing courses with focus on digital skills would have a good potential to increase engagement with active T2D self-care by using eHealth in this high-risk group.

## Appendix

Multimedia Appendix 1 Complete summary of studies shown in Tables 1-3.

## Abbreviations

eHealth: electronic health
ICT: information and communication technology
NS: not significant
PC: principal component
SNS: social network service
T1D: type 1 diabetes
T2D: type 2 diabetes
