# Correction: The Association Between Commonly Investigated User Factors and Various Types of eHealth Use for Self-Care of Type 2 Diabetes: Case of First-Generation Immigrants From Pakistan in the Oslo Area, Norway

**DOI:** 10.2196/11888

**Published:** 2018-08-31

**Authors:** Naoe Tatara, Hugo Lewi Hammer, Hege Kristin Andreassen, Jelena Mirkovic, Marte Karoline Råberg Kjøllesdal

**Affiliations:** ^1^ Department of Computer Science Faculty of Technology, Art and Design Oslo and Akershus University College of Applied Sciences Oslo Norway; ^2^ Centre for Care Research Norwegian University of Science and Technology Gjøvik Norway; ^3^ Norwegian Centre for E-health Research University Hospital of North Norway Tromsø Norway; ^4^ Center for Shared Decision Making and Collaborative Care Research Oslo University Hospital Oslo Norway; ^5^ Department of Community Medicine and Global Health, Institute of Health and Society Faculty of Medicine University of Oslo Oslo Norway

The authors of “The Association Between Commonly Investigated User Factors and Various Types of eHealth Use for Self-Care of Type 2 Diabetes: Case of First-Generation Immigrants From Pakistan in the Oslo Area, Norway” (JMIR Public Health Surveill 2017;3(4):e68) would like to make changes to the following areas in the Results section:

1. Table 6
  - The label “(h) Keeping track of health information” should be replaced with “(i) Self-assessment of health”.
  - The label “(i) Self-assessment of health” should be replaced with “(h) Keeping track of health information”.
  - Heading of column “Log odds ratio” should be replaced with “Estimate”.
2. In the “Association Between User Factors and eHealth Use” sub-section, in the last paragraph, the second last sentence is “The *health* component is negatively related to closed online communication about T2D with a few acquaintances (d), and there is an indication of a positive relation between the *health* component and the use of Web applications and mobile apps for active decision making on T2D self-care by self-assessing of health status (*P*=.05).” This should be replaced with: “The *health* component is negatively related to closed online communication about T2D with a few acquaintances (d), and there is an indication of a positive relation between the *health* component and the use of Web applications and mobile apps for active decision making on T2D self-care by tracking of health information (*P*=.05).”

The errors were caused by an inadvertent mistake on labeling results of statistical analysis before drafting the manuscript and the oversight of the missing label for the result of the Poisson regression analysis. As the results of both logistic regression analysis and Poisson regression analysis are presented in the same table, the label “Estimate” should be used to express the results in the most appropriate manner.

Although the errors concern changes in results, the changes do not impact on the conclusion.

Regarding the missing label for the result of the Poisson regression analysis, the method is clearly stated in the Methods section, and thus we consider that the impact of this change is minor.

The correction will appear in the online version of the paper on the JMIR website on August 27, 2018, together with the publication of this correction notice. Because this was made after submission to PubMed, Pubmed Central, and other full-text repositories, the corrected article also has been re-submitted to those repositories.

