# Supplementary material for: The Association Between Commonly Investigated User Factors and Various Types of eHealth Use for Self-Care of Type 2 Diabetes: Case of First-Generation Immigrants From Pakistan in the Oslo Area, Norway
Source: JMIR Public Health Surveill. 2017 Oct 5;3(4):e68. doi: 10.2196/publichealth.7009 (PMC5649041; doi:10.2196/publichealth.7009)
Supplement: Multimedia Appendix 1 [file publichealth_v3i4e68_app1.pdf]

Appendix 1 Complete summary of studies shown in Tables 1-3

Studies listed in Table 1

First author and the year of publication	Data taken in	Country	Description of eHealth use	Sample	Inclusion of immigrants or ethnic minority informants	Statistical analysis method
Kalantzi, 2015 [29]	2012	Greece	Internet usage as an important information source for DM (28.1% has used Internet as an information souce of DM, but only 5.4% considered it important)	Patients being followed up at the outpatient Diabetes Clinic and the outpatient Diabetes Foot Clinic of the univ. Affiliated Laiko General Hospital in Athens, either Type 1 or Type 2 diabetes, Age >18, Understanding Greek to answer the question, N=203	No info	Categorical variables: Chi-square test<br>Ordinal data: Kendall's tau test
Lee, 2012 [31]	Unknown	US, NYC	Using Internet to seek health or medical information (14.5% of the sample reported Health-Information Seeking Behaviors and most respondents were female)	Hispanics in Washington Height and Inwood area, N=100	Hispanics	Binary logistic regression
Mesch, 2012 [36]	2010	Israel	Searching for health information: How often they had searched for information on the Internet about vaccines, high blood pressure, doctors, quitting smoking, diet, healthy food and protection from the sun, with 5-point Likert scale (1: never, 5: very frequently) ("For those with access to the Internet (68.3% of the totally 2000 respondents), 62.3% reported searching for health and medical information online")	"A large representative sample of the population was extracted, and 4000 phone numbers were contacted with a response rate of 50%". Subset of Internet users, Avr. Age = 43.04 (SD=15.81), N=1371.	Immigrants from the former Soviet Union (n=252)<br>Israeli Arab (n=367)<br>Native Israelis (n=752)	Ordinary Least Square regression

Horizontally continued from the previous page

First author and the year of publication	Education level	Age	Female	Disease related factors
Kalantzi, 2015 [29]	(+) (Details not shown)	(-) (Details not shown)	N.S. (Not clearly written, but considered so from Table 5 and the text)	(+) Having T1DM<br>Lower duration of the disease<br>Being followed-up in the Diabetes Clinic (vs. Diabetes Foot Clinic)<br>Having better DM control (Details not shown)
Lee, 2012 [31]	(+) (Details not shown)	(-) (Details not shown)	None	(+) Better general health status (Details not shown)
Mesch, 2012 [36]	(-) College (beta = -0.08, p < .01), Graduate school (beta = -0.08, p < .01) (Ordinary Least Squares analysis, Negative association (Beta<0))	(-) (beta = -0.19, p < .01)	(+) (On the paper, male = 1, and beta = -0.09, p < .01)	Hypertension (beta = 0.06, p < .05)<br>Heart disease (beta = 0.07, p < .01)<br>Patient satisfaction with physician (beta = 0.08, p < .01)<br><br>N.S: Diabetes, Cancer

Horizontally continued from the previous page

First author and the year of publication	Income	Others
Kalantzi, 2015 [29]	(+) (Details not shown)	
Lee, 2012 [31]	None, but employment status (N.S., Details not shown)	
Mesch, 2012 [36]	N.S.	(-) Immigrants (beta = -0.16, p < .01) (vs. Israeli Arabs (Ref)), Israeli Jews (beta = -0.24, p < .01) (vs. Israeli Arabs (Ref))<br><br>N.S.: (Marital status, Number of children, Frequency of Internet use)

Studies listed in Table 1

First author and the year of publication	Data taken in	Country	Description of eHealth use	Sample	Inclusion of immigrants or ethnic minority informants	Statistical analysis method
Gonzalez, 2016 [24]	2011-2012	USA, CA	health information-seeking behavior in the last 12 months, defined as seeking health or medical information online, including information about disease symptoms, diet or nutrition, physical activity, health care providers, and health insurance plans (3190 (53.36% of the Internet users) had such experience)	California Health Interview Survey 2011-2012, representative sample of noninstitutionalized California state adult population, age >18, (Latinos and non-Hispanic whites, N=27289, Latinos n=9506, Lations using the Internet for the last 12 months n=6037)	Latinos (n=9506, among which Internet users in the last 12 months, n=6037)	Logistic regression
Wangberg, 2015 [9]	2013	Norway	Read about diet and exercise (61.22% had such experience)	Web panel, age > 15, stratified, N=1028	No info	Chi-square test
Manierre, 2015 [34]	2011, 2013	USA	(Among Internet users) Has looked for health information on the Internet for self or someone else in the past 12 months? (Unclear from the tables (2 and 3))	Health Information National Trends Survey (HINTS) data from 2011 and 2013 (Model 4) (n = 5510)	Hispanic<br>White<br>Black<br>Asian<br>Other/Multiple	Logistic regression

Horizontally continued from the previous page

First author and the year of publication	Education level	Age	Female	Disease related factors
Gonzalez, 2016 [24]	(+) (below high school: OR 0.33, 95% CI 0.24-0.46, P<.001; high school: OR 0.53, 95% CI 0.41-0.67, P<.001; some college: OR 0.72, 95% CI 0.59-0.87, P=.001, ref: bachelor or higher)	(-) (aged 65 years or older, (ref: aged 18-24) (OR 0.54, 95% CI 0.40-0.75, P<.001))	(+) (OR 1.50, 95% CI 1.27-1.77, P<.001)	(+) Chronic diseases, binge drinks once a month or more often<br>(-) smoker<br><br>N.S. (obese, drink soda >= 2 times/week, Eats >=2 servings of fast food per week, Eats <7 servings of vegetables per week, Walks <150 min per week)
Wangberg, 2015 [9]	(+) (chi-squared: 19.1, p <.001)	None	(+) (chi-squared: 25.6, p < .001)	None
Manierre, 2015 [34]	None	None	(+) (OR: 1.82, P < .001)	N.S. (Self-rated health "poor" against "excellent" (+) and "has/had cancer" (+), among female, HINTS 2003 and 2005)<br><br>(Partial mediating effect: Perceived cancer risk, i.e. men tend to have lower levels of perceived cancer risk compared to women)

Horizontally continued from the previous page

First author and the year of publication	Income	Others
Gonzalez, 2016 [24]	(+) (Poverty level, Poor (0-99% of federal porverty level (FPL)): OR 0.75, 95% CI 0.61-0.93, P=.01, ref: above poor (≥ 200% of the FPL))	(+) Has usual sources of care (OR 1.43, 95% CI 1.14-1.79, P=.01)<br><br>(-) Foreign born Latinos (OR 0.71, 95% CI 0.58-0.88, P=.002, compared to US-born Latinos)
Wangberg, 2015 [9]	None	
Manierre, 2015 [34]	N.S.	

Studies listed in Table 1

First author and the year of publication	Data taken in	Country	Description of eHealth use	Sample	Inclusion of immigrants or ethnic minority informants	Statistical analysis method
Lee, 2014 [48]	2011-2012	US	In the last 12 months Either:<br>1) Participated in an online support group for people with similar health or mdeical issues<br>2) Have used email or the Internet to communicate with a doctor or doctr's office<br>3) Have used the Internet to look up health or medical information<br>(In total 7.89% had such experience)	Hispanic, Age >18, English or Spanish speaking, Washington Heights Inwood community of Northern Manhattan, US, N=4070	Hispanics	Binary logistic regression
Kontos, 2014 [5]	2011-2012	USA	In the last 12 months, used the Internet to download health-related info to a mobile device (11.07% (Unweighted%) had such experience)<br><br>In the last 12 months, used the Internet to look for health or medical information for self (77.7% (Unweighted%) had such experience)	HINTS 4 Cycle 1, 2012, N=2358 respondents identified as Internet users	Non-Hispanic white<br>Hispanic<br>Non-Hispanic black<br>Non-Hispanic other	Multivariable logistic regression
AlGhamdi, 2015 [17]	2010	Saudi Arabia	Used the Internet to search for health-related information (58.4% (363/622) had such experience).	Outpatients and visitors at the waiting area of the general pharmacy at the King Khalid Univ. Hospital, Riyadh, N=801 inc. Missing data of some questions (response rate: 80.1%)	No info	Multiple logistic regression

Horizontally continued from the previous page

First author and the year of publication	Education level	Age	Female	Disease related factors
Lee, 2014 [48]	(+) (OR: 3.03, 95%CI: 2.15-4.29, P < .001)	N.S.	N.S.	(-) General health status (OR: 0.42 95%CI: 0.31-0.57, P < .001)<br>Not having hypertension
Kontos, 2014 [5]	(?) (significant difference between "college degree or more" (ref) and "some college" (OR: 0.54, 95%CI: 0.33-0.89), but not between "college degree or more" and "high school degree or less")	N.S.	N.S.	None
	N.S.	(-) (Significant difference between 18-34 (OR:3.51, 95%CI:1.66-7.44) and >65 (ref), and between 35-49 (OR:2.35, 95%CI:1.17-4.72) and >65, but N.S. between 50-64 and >65)	N.S.	None
AlGhamdi, 2015 [17]	(+) (significant difference between university or higher and High school or lower (ref) (OR: 1.7, 95%CI: 1.1-2.8))	(?) (significant difference between 30-39 and ≥40 (ref) (OR: 2.0, 95%CI: 1.1-3.7, N.S: between 20-29 and ref, and between <20 and ref)	(+) OR:3.8, 95%CI: 2.3-6.4	N.S. (Medical condition (temporary vs. chronic (ref), Severity of disease (mild, moderate, vs. Severe (ref), No. Of visits to doctor (Once, 2-5 times, vs. More than 5 times (ref.))

Horizontally continued from the previous page

First author and the year of publication	Income	Others
Lee, 2014 [48]	None, but employment status or insurance (both N.S.)	(+) Use of SNS (OR: 3.78, 95%CI: 2.78-5.13, P < .001)<br><br>N.S. (Health literacy level (NVS score))
Kontos, 2014 [5]	N.S.	(+) Other race (OR: 2.78, 95%CI: 1.33-5.86, Ref: Non-Hispanic white)<br>N.S.: Hispanic, Non-Hispanic black, Non-Home ownership, Born outside of US
	N.S.	N.S.: Hispanic, Non-Hispanic black, or Other race (Ref: Non-Hispanic white), Non-Home ownership, Born outside of US
AlGhamdi, 2015 [17]	(+) (Significant difference between >15,000 (SR, 3.74 SR = 1USD) and ≤10,000 (ref) (OR: 2.8, 95%CI: 1.5-5.1) N.S: between 10,000 - 15,000 and ref.)	(?) Occupation (Employed vs. Unemployed (ref.) OR: 2.7, 95%CI: 1.4-4.9, N.S.: Private business, student vs. Ref)<br><br>N.S.: Marital status

Studies listed in Table 1

First author and the year of publication	Data taken in	Country	Description of eHealth use	Sample	Inclusion of immigrants or ethnic minority informants	Statistical analysis method
Bjunowska-Fedak, 2015 [20]	2012	Poland	Used the Internet to obtain information about health or illness (81%, 62/77 Internet users)	Age ≥ 60, Polish, N=242, Internet users: n=77	None	Multivariable analysis (Comparison of average scores for each factor between Internet users for health (n=62) and Internet non-users for health (n=180), t-test for normally distributed variables with equal variances in groups, Mann-Whitney Utest for non-normally distributed variables, chi-squared test for qualitative varaibles)
Bjunowska-Fedak, 2015 [21]	2012 (data taken in 2005 and 2007 are also shown)	Poland	Frequency of using the Internet to get information about health or illness (66.7% of the sample, and 80.8% of the Internet users were categorized as Internet users for health purpose)	Random digital dialing to adults aged 18-80+, Sample size for 2012 was N=1000.	No info	Wilcoxon rank order test for unrelated sample for quantitaive variables, chi-square test for qualitative variables.<br>Estimates of effect size: the difference in location for quantitative variables, Cramer's V for qualitative variables, and CI for both.

Horizontally continued from the previous page

First author and the year of publication	Education level	Age	Female	Disease related factors
Bjunowska-Fedak, 2015 [20]	(+) (users: A level 30.6%, B level 40.3%, C level 29.1%; non-users: A level 54.4%, B level 31.7%, C level 13.9%) ((A) education level lower than upper secondary; (B) education level including upper secondary to post-secondary non-tertiary; and (C) education level covering all levels according to ISCED higher than post-secondary non-tertiary)	(-) (users: 66.0±5.6, non-users: 71.8±8.4)	N.S.	N.S.: (Health status (subjective assessment), Frequency of doctor's visits during the last 12 months, Chronic diseases/didsability)
Bjunowska-Fedak, 2015 [21]	(+) (Internet Users for Health Purpose: Lower than upper secondary: 24.4%; upper secondary to post-secondary non-tertiary: 38.1%; higher than post-secondary non-tertiary: 37.5%, Internet Non-Users for Health Purpose: Lower than upper secondary: 41.1%; upper secondary to post-secondary non-tertiary: 24.7%; higher than post-secondary non-tertiary: 34.2%,P = .007, Estimate of effect size: 0.12, 95%CI: 0.04-0.19)	(-) (Internet Users for Health Purpose: Mean: 38.73, SD: ±14.20, Median: 37, Min-Max: 18-83, Internet Non-Users for Health Purpose: Mean: 46.59, SD: ±15.92, Median: 47, Min-Max: 20-91,,P < .001, Estimate of effect size: -8, 95%CI: -12 - -4)	(+) (Internet Users for Health Purpose: Men: 47.4%, Women: 52.6%, Internet Non-Users for Health Purpose: Men 78.1%, Women: 21.9%, P < .001, Estimate of effect size: 0.18, 95%CI: 0.11-0.25)	(+) Frequency of doctor's visits during the last 12 months (Internet Users for Health Purpose: Mean: 5.60, SD: ±9.72, Median: 3, Min-Max: 0-99, Internet Non-Users for Health Purpose: Mean: 4.44, SD: ±7.17, Median: 2, Min-Max: 0-45,P = .0032, Estimate of effect size: 1.0, 95%CI: 0.0 - 1.0)<br>N.S.: (Health status (subjective assessment), Chronic diseases/didsability)

Horizontally continued from the previous page

First author and the year of publication	Income	Others
Bjunowska-Fedak, 2015 [20]	None	(+) Living with family (users: alone 22.6%, with family 77.4%; non-users: alone: 35.6%, with family 64.4%), Mobile phone use (users: yes 88.7%, no 11.3%; non-users: yes 66.1%, no 33.9%)<br><br>N.S.: Employment status, residence
Bjunowska-Fedak, 2015 [21]	N.S.: Employment status, Residence	N.S.: Mobile phone use

Studies listed in Table 1

First author and the year of publication	Data taken in	Country	Description of eHealth use	Sample	Inclusion of immigrants or ethnic minority informants	Statistical analysis method
Duplaga, 2013 [23]		Poland	Declaration of the Internet as one of main sources of health-related information (29.7% (n=49) of all respondents and 47.9% (n = 46) of those who were Internet users answered so.)	Patients admitted to the Department of Pulmonology, Jagiellonian University Medical College, Krakow, Poland or attending the pulmonary polyclinics at the department, with an established diagnosis of a chronic disease with an established treatment	No info	Fisher's exact test (Sex, Bronchial asthma, COPD)<br>Pearson's chi-squared test (Age, Place of residence, Education, Duration of chronic disease, Hospitalization due to chronic disease)
Beck, 2014 [18]		France	Having used the Internet during the last 12 months to look for either information or advice on health (474/977 Web users, 48.5% had such experience)	Young adults aged 15-30 years, N = 1052	No info	Multivariate logistic regression
Nölke, 2015 [38]	2011	Germany (Northe Rhine-Westphali a (NRW))	Use the Internet to search for information on medical or health issues (67.9% (/1488) answered yes)	NRW residents with landline telephone, age ≥ 18, speaking German, Internet users, telephone survey, N=1488 (For final multivariate model, n=1002)	Own or parental migration background (no information about country of origin)	Multiple logistic regression (Final multivariate model n = 1002, R^2 = 0.151)

Horizontally continued from the previous page

First author and the year of publication	Education level	Age	Female	Disease related factors
Duplaga, 2013 [23]	(+) (Lower than upper secondary: 18.0%; upper secondary to post-secondary non-tertiary: 39.6%; higher than post-secondary non-tertiary: 35.2%, P = .03)	(-) (<39: 51.3%; ≥39 to <52: 34.2%; ≥52 to <61: 22.0%; ≥61; 15.0%, P=.003)	N.S.	COPD (-) (N.S.: Duration of chronic disease, hospitalization due to chronic disease, bronchial asthma)
Beck, 2014 [18]	None	(+) (15-19 years: 39.3%, 20-25 years: 50.4%, and 26-30 years: 55.4% (P=.002))	(conditional +) Women with psychological distress (OR: 2.0, 95%CI: 1.0-4.0), pregnant women or women with at least 1 child (OR: 1.8, 95%CI: 1.1-2.7)	(+) Women with psychological distress (OR: 2.0, 95%CI: 1.0-4.0)<br>N.S.: Having a chronic disease
Nölke, 2015 [38]	(+) (Social class*: Significant difference between middle and lower (ref) (OR:2.24, 95%CI: 1.56-3.20), Upper and ref. (OR:4.04, 95%CI: 2.65-6.17)	N.S.	(+) OR:1.52, 95%CI: 1.12-2.08	(+) Use of health care services (significant difference between high and none (ref.) OR:1.73, 95%CI: 1.19-2.51,<br>N.S.: Use of health care services, Low vs. ref.; health insurance status, self-perceived health, chronic diseases/conditions)

* Social class: (citation from the paper) "The independent variable 'social class' was generated based on Winkler's social class index, comprising the three socio-economic dimensions 'educational qualification', 'occupational status' and 'household net income'. Calculating the index, points on a scale from 1 to 7 were assigned for each dimension, 1 being the lowest and 7 the highest educational qualification/occupational status/household income group. The points from each dimension were then summed to yield a total score. Depending on the score, respondents were classified as belonging to the lower, middle or upper social class."

Horizontally continued from the previous page

First author and the year of publication	Income	Others
Duplaga, 2013 [23]	None	N.S: Place of residence
Beck, 2014 [18]	N.S.: Socio-occupational status, Quality of life	
Nölke, 2015 [38]	(+) (Social class: Significant difference between middle and lower (ref) (OR:2.24, 95%CI: 1.56-3.20), Upper and ref. (OR:4.04, 95%CI: 2.65-6.17)	(-) Migration background (Significant difference between Own migration background and no migration background (ref.) OR:0.56 95%CI: 0.38-0.82)<br><br>N.S: Parental migration background, parental status, employment status, town size

Studies listed in Table 2

First author and the year of publication	Data taken in	Country	Description of eHealth use	Sample	Inclusion of immigrants or ethnic minority informants	Statistical analysis method
Mesch, 2012 [36]	2010	Israel	Communicating about health: The extent to which they had participated in Internet forums about health issues or sent and e-mail to a physician or a nurse, with a 5-point Likert scale (1: never, 5: very frequently) (Information about percentage of the informants who had such experience is missing)	"A large representative sample of the population was extracted, and 4000 phone numbers were contacted with a response rate of 50%". Subset of Internet users, N=1371.	Immigrants from the former Soviet Union (n=252)<br>Israeli Arab (n=367)<br>Native Israelis (n=752)	Ordinary Least Square regression
Wangberg, 2015 [9]	2013	Norway	Asked questions about exercise or diet to prof. (6.72% had such experience)<br><br>Posted a status about exercise or diet on SNS (17.45% had such experience)<br><br>Shared online exercise or diet data with others (7.55% had such experience)<br><br>Discussed exercise or diet with peers (9.67% had such experience)	Web panel, age > 15, stratified, N=1028	No info	Chi-square test

Horizontally continued from the previous page

First author and the year of publication	Education level	Age	Female	Disease related factors
Mesch, 2012 [36]	N.S.	N.S.	N.S.	Patient satisfaction with physician (+) (beta = 0.10, p < .01)<br><br>N.S. (Hypertension, Heart disease, Diabetes, Cancer)
Wangberg, 2015 [9]	(-) (Primary: 19/150 (12.7%), Secondary: 13/561 (2.3%), Tertiary: 18/300 (6.0%), chi-squared: 10.5, p = .005)	None	N.S.	None
	(+) (Primary: 21/149 (14.1%), Secondary: 87/558 (15.6%), Tertiary: 68/297 (22.9%), chi-squared: 8.58, p <.014)	None	(+) (Women: 114/504 (22.62%), Men: 61/449 (12.22%), chi-squared: 18.8, p < .001)	None
	N.S.	None	(-) (Women: 33/503 (6.56%), Men: 43/504 (8.53%), chi-squared: 13.4, p < .001)	None
	N.S.	None	N.S.	None

Horizontally continued from the previous page

First author and the year of publication	Income	Others
Mesch, 2012 [36]	N.S.	(-) Immigrants (beta = -0.13, p < .01) (vs. Israeli Arabs), Israeli Jews (beta = -0.18, p < .01) (vs. Israeli Arabs)<br><br>N.S.: Marital status, Number of children, Frequency of Internet use
Wangberg, 2015 [9]	None	
	None	
	None	
	None	

Studies listed in Table 2

First author and the year of publication	Data taken in	Country	Description of eHealth use	Sample	Inclusion of immigrants or ethnic minority informants	Statistical analysis method
Kontos, 2014 [5]	2011-2012	USA	In the last 12 months, used email or Internet to comunicate with a doctor or doctor's office (21.59% (Unweighted%) had such experience)<br><br>In the last 12 months, participated in an online support group for people with a similar health or medical issue (4.66% (Unweighted%) had such experience)<br><br>In the last 12 months, visited a social networking site to read and share about medical topics (14.63% (Unweighted%) had such experience)	HINTS 4 Cycle 1, 2012, N=2358 respondents identified as Internet users	Non-Hispanic white<br>Hispanic<br>Non-Hispanic black<br>Non-Hispanic other	Multivariable logistic regression

Horizontally continued from the previous page

First author and the year of publication	Education level	Age	Female	Disease related factors
Kontos, 2014 [5]	(+) (significant difference between "college degree or more" (ref) and "high school degree or less" (OR: 0.50, 95%CI: 0.33-0.76), but not between "college degree or more" and "some college")	(-) (Significant difference between 18-34 (OR:2.24, 95%CI:1.20-4.16) and >65 (ref), but N.S. between 35-49 and >65, and between 50-64 and >65)	(+) (OR:1.53 95%CI: 1.14-2.04)	None
	N.S.	N.S.	(+) (OR:2.79 95%CI: 1.20-6.51)	Family history of cancer (OR: 2.96 95%CI: 1.00-3.83)<br>N.S.: Cancer diagnosis (self)
	? (significant difference between "college degree or more" (ref) and "some college" (OR: 1.59, 95%CI: 1.06-2.39), but not between "college degree or more" and "high school degree or less")	(-) (Significant difference between 18-34 (OR:2.81, 95%CI:1.13-7.00) and >65 (ref), and between 35-49 (OR:2.27, 95%CI:1.00-5.17) and >65, but N.S. between 50-64 and >65)	N.S.	N.S. (Cancer diagnosis (self) and family history of cancer)

Horizontally continued from the previous page

First author and the year of publication	Income	Others
Kontos, 2014 [5]	N.S.	Other race (vs. Non-Hispanic white)(OR: 2.02 95%CI:1.01-4.04)<br>N.S.: Hispanic, or Non-Hispanic black (vs. Non-Hispanic white), Non-Home ownership, Born outside of US
	(?) (Significant difference between $50,000-<75,000 vs. >$75,000 (ref), OR: 2.22 95%CI:1.04-4.73)	N.S.: Hispanic, Non-Hispanic black, or Other race (vs. Non-Hispanic white), Non-Home ownership, Born outside of US
	(-) (Significant difference between <$20,000- vs. >$75,000 (ref), OR: 2.12 95%CI:1.04-4.29)	N.S.: Hispanic, Non-Hispanic black, or Other race (vs. Non-Hispanic white), Non-Home ownership, Born outside of US

Studies listed in Table 2

First author and the year of publication	Data taken in	Country	Description of eHealth use	Sample	Inclusion of immigrants or ethnic minority informants	Statistical analysis method
Tennant, 2015 [42]	2013	USA	In last 12 months, have you used the Internet for any of the following reasons to locate or share health information?" Respondents could select all reasons for using the Internet: (1) participated in a Web-based-support group, (2) used a social networking site like Facebook/Twitter/ LinkedIn, or (3) wrote in a Web-based diary or blog (35.7%, 101/283, had such experience. (1) 11/101, 10.9%, (2) 96/101, 95.0%, (3) 6/101, 5.9%, 90/101, 89.1% had used only one type of them)	Age ≥ 50, Internet (or email) users, N=283	White (n = 252)<br>Black (n = 10)<br>Asian or Pacific Islander (n = 1)<br>American Indian or Alaska native (n = 3)<br>Other (n = 6)<br>Multi-racial or mixed race (n = 7)<br>Non response (n=4)	Multiple logistic regression

Horizontally continued from the previous page

First author and the year of publication	Education level	Age	Female	Disease related factors
Tennant, 2015 [42]	(+) (significant difference between post graduate and < high school (ref) (OR: 7.11, 95%CI: 1.11-45.56), 4 years of college and ref. (OR: 2.57, 95%CI: 1.00-6.59)), N.S.: Some college, High school graduate	N.S.	(+) (OR: 2.63 95%CI: 1.35-5.13)	N.S. (Health status)

Horizontally continued from the previous page

First author and the year of publication	Income	Others
Tennant, 2015 [42]	N.S.	(+) Use of one (OR: 3.68 95%CI: 1.56-8.68) or more (OR: 6.06 95%CI: 2.50-14.69) electronic device for health information<br><br>N.S.: ethnicity, race, marital status

Studies listed in Table 2

First author and the year of publication	Data taken in	Country	Description of eHealth use	Sample	Inclusion of immigrants or ethnic minority informants	Statistical analysis method
Thackeray, 2013 [43]	2010	USA	Using SNS for health-related activities inc. (1) get health information, (2) start or join a health-related group, (3) follow your friend's personal health experiences or health updates, (4) raise money or draw attention to a health-related issue or cause, and (5) remember or memorialize others who suffered from a certain health condition (Pew questions Q26a-e). The composite scale had an internal reliability of Cronbach alpha=.66 (551/1745, 31.58% had such experience)	US residents, age ≥ 18, English speaking, random digit methods, telephone survey, N = 1745	White (n = 1382)<br>Black or African American (n = 195)<br>All other races (n = 136)<br>Answer not given (n = 32)	Multivariate logistic regression analysis.

Horizontally continued from the previous page

First author and the year of publication	Education level	Age	Female	Disease related factors
Thackeray, 2013 [43]	N.S.	(-) (Adjusted OR: 0.96, 95%CI: 0.95-0.97)	(+) (Male vs. Female (ref.), Adjusted OR: 0.70 95%CI: 0.56-0.87)	N.S: (chronic health condition)

Horizontally continued from the previous page

First author and the year of publication	Income	Others
Thackeray, 2013 [43]	N.S.	(+) Having a regular health care provider (Adjusted OR: 1.89, 95%CI: 1.43-2.52)<br><br>N.S.: Marital status, geographic community type, health insurance, race

Studies listed in Table 3

First author and the year of publication	Data taken in	Country	Description of eHealth use	Sample	Inclusion of immigrants or ethnic minority informants	Statistical analysis method
Krebs, 2015 [14]	2015	US	Have ever downloaded an "app" to track anything related to a user's own health (58.23% had such experience)	English speaking, Mobile phone owners, age > 18, stratified sampling to achieve balanced diversity of informants in terms of gender, income, education and race. N = 1604	African American or black<br>Asian American or Asian<br>White or Caucasian<br>Native American / Pacific islander<br>Latino/Hispanic	Multivariable Poisson regression (GENMOD procedure in SAS, using methods outlined by Zhao 2013, "Proper estimation of relative risk using PROC GENMOD in population studies")
Bender, 2014 [15]	2013	US	Download health apps (19.8% (175/904) had such experience)	N=904<br>Caucasian (n=172), Filipino (n=250), Korean (n=234), Latino (n=248), age > 18, no history of diabetes.	Caucasian<br>Filipino<br>Korean<br>Latino	Backward stepwise multiple logistic regression
Wangberg, 2015 [9]	2013	Norway	Used Internet- or mobile-based programs to support health behavior (16.90% had such experience)<br><br>Kept an online exercise or diet journal (17.87% had such experience)	Web panel, age > 15, stratified, N=1028	No info	Chi-square test

Horizontally continued from the previous page

First author and the year of publication	Education level	Age	Female	Disease related factors
Krebs, 2015 [14]	(+) (RR: 1.12, 95%CI: 1.03-1.22, vs less than high school)	(-) (RR: 0.98 95%CI: 0.97-0.98, each year)	N.S.	N.S. (Diagnosed with chronic disease, BMI)
Bender, 2014 [15]	(+) (Significant difference between; college or some college vs. high school or some high school (Adjusted OR: 2.62, 95%CI: 1.44-4.80), Graduate school vs. high school or some high school (Adjusted OR: 2.93, 95%CI: 1.43-6.0))	(-) (Each year, Adjusted OR: 0.96 95%CI: 0.95-0.97)	N.S.	N.S. (BMI, smokes cigarettes, high BP, high chol., family member with DM, family member with heart attack, physical inactivity, perceived risk for MI, self-reported health status, discussed diabetes with provider)
Wangberg, 2015 [9]	N.S.	None	(+) (Women: 102/505 (20.20%), Men:68/501 (13.57%), chi-squared: 7.9, p = .005)	None
	(+) (Primary: 23/148 (15.5%), Secondary: 82/558 (14.7%), Tertiary: 75/300 (25.0%), chi-squared: 19.1, p <.001)	None	(+) (Women: 106/503 (21.07%), Men:74/504 (14.68%), chi-squared: 7.0, p =.008, Significance dissappeared by adding independent variable of "More satisfied with appearance")	Good or very good subjective health (+)

Horizontally continued from the previous page

First author and the year of publication	Income	Others
Krebs, 2015 [14]	(+) (Significant difference between US $100,000 + vs. US $50,000 - 74,999 (RR: 1.33 95%CI: 1.16 - 1.51), US $75,000-99,999 vs. US $50,000 - 74,999 (RR: 1.32 95%CI: 1.16 - 1.50), and < US $25,000 vs. US $50,000 - 74,999 (RR: 0.79 95%CI: 0.67 - 0.93)	(+) Latino / Hispanic (vs. White, RR:1.12 95%CI: 1.03-1.22)<br>N.S.: African American or black, Asian American or Asian (vs. White)
Bender, 2014 [15]	None	(+) Family member with myocardial infarction (Adjusted OR: 2.02, 95%CI: 1.16-3.51)<br><br>(-) Korean vs. Caucasian (Ref.) (Adjusted OR: 0.52, 95%CI: 0.31-0.88), Latino vs. Caucasian (Ref.) (Adjusted OR: 0.37, 95%CI: 0.20-0.69)), Paper survey vs. Online survey (Adjusted OR: 0.50, 95%CI: 0.34-0.75)<br><br>N.S.: Filipino vs. Caucasian (Ref.), Marital status, years lived in the US
Wangberg, 2015 [9]	None	
	None	

Studies listed in Table 3

First author and the year of publication	Data taken in	Country	Description of eHealth use	Sample	Inclusion of immigrants or ethnic minority informants	Statistical analysis method
Kontos, 2014 [5]	2011-2012	USA	In the last 12 months, used the Internet to keep track of personal health information (21.25% (Unweighted%) had such experience)<br><br>In the last 12 months, used a website to help with diet, weight, or physical activity (39.23% (Unweighted%) had such experience)	HINTS 4 Cycle 1, 2012, N=2358 respondents identified as Internet users	Non-Hispanic white<br>Hispanic<br>Non-Hispanic black<br>Non-Hispanic other	Multivariable logistic regression

Horizontally continued from the previous page

First author and the year of publication	Education level	Age	Female	Disease related factors
Kontos, 2014 [5]	(+) (significant difference between "college degree or more" (ref) and "high school degree or less" (OR: 0.53, 95%CI: 0.32-0.84), but not between "college degree or more" and "some college")	N.S.	(+) (OR: 1.52, 95%CI: 1.06-2.19)	None
	(+) (significant difference between "college degree or more" (ref) and "high school degree or less" (OR: 0.64, 95%CI: 0.42-0.98), and between "college degree or more" and "some college" (OR: 0.67, 95%CI: 0.49-0.93))	(-) (Significant difference between >65 (ref) and; 18-34 (OR:3.37, 95%CI:2.00-5.69); 35-49 (OR:2.57, 95%CI:1.66-3.99); and 50-64 (OR:2.22, 95%CI:1.43-3.43))	N.S.	None

Horizontally continued from the previous page

First author and the year of publication	Income	Others
Kontos, 2014 [5]	N.S.	N.S.: Hispanic, Non-Hispanic black, or Other race (vs. Non-Hispanic white), Non-Home ownership, Born outside of US
	N.S.	Other race (vs. Non-Hispanic white)(OR: 2.78 95%CI:1.33-5.86)<br>N.S.: Hispanic, or Non-Hispanic black (vs. Non-Hispanic white), Non-Home ownership, Born outside of US
